# Efficacy of Platelet Rich Fibrin in Myringoplasty

**DOI:** 10.12669/pjms.37.1.3059

**Published:** 2021

**Authors:** Nida Riaz, Muhammad Ajmal, Muhammad Sheharyar Khan

**Affiliations:** 1 Dr. Nida Riaz, FCPS. Department of Otorhinolaryngology and Head and Neck Surgery, Holy Family Hospital, Rawalpindi, Pakistan; 2 Dr. Muhammad Ajmal, FRCS. Department of Otorhinolaryngology and Head and Neck Surgery, Holy Family Hospital, Rawalpindi, Pakistan; 3 Dr. Muhammad Sheharyar Khan, MBBS. House Officer, Rawalpindi Medical University, Rawalpindi, Pakistan

**Keywords:** Myringoplasty, Platelet rich fibrin, Tympanic membrane, Temporalis fascia graft

## Abstract

**Objective:**

To determine the efficacy of usage of topical autologous platelet rich fibrin (PRF) in improving outcomes of myringoplasty regarding graft uptake and hearing improvement.

**Methods:**

This study was conducted in the ENT department of Holy Family Hospital, Rawalpindi, over a period of six months from August 2019 to January 2020. It was designed as a prospective single blinded randomized controlled trial. The study involved a total of 50 participants in whom myringoplasty was done through post auricular approach using underlay technique. In 25 patients topical drops of PRF were used. Outcomes were compared after three months with the control group (n=25), who underwent myringoplasty without PRF.

**Results:**

After three months follow-up, graft uptake was reported 78% and 52% in cases and controls, respectively (P=0.070). Mean hearing improvement was 18 dB and 6 dB in cases and controls, respectively (P=0.014). Postoperative infection occurred in 8% of the cases, and in 32% controls (P=0.037).

**Conclusion:**

Topical use of Platelet-Rich Fibrin during myringoplasty results in improved graft uptake. Hence, resulting in much improved hearing, significant reduction in infection rates and decrease in perforation sizes.

## INTRODUCTION

Myringoplasty is surgical closure of tympanic membrane perforations, without reconstruction of the ossicular chain.^1^ It is an established procedure; regarded as the most effective method of tympanic membrane closure .Most commonly used techniques of myringoplasty are overlay and underlay.^2^ Different varieties of grafts like skin, fascia, fat, vein, perichondrium and dura matter have been employed^3^. Temporalis fascia is considered as the most preferred graft material.^4^

Usage of bioactive agents to improve healing has become common and has remarkably improved surgical outcomes. Different bioactive agents have been used including; hyaluronic acid, fibrin sealants, platelet rich plasma, pentoxifylline, fibroblast growth factors etc^5^-^7^. PRF (platelet rich fibrin) is basically plasma enriched with higher concentration of autologous human platelets^8^. Degradation of granules within the PRF and release of growth factors is the basic mechanism of enhanced healing.^9^

Usage of all these techniques has a reasonable impact on the hearing improvement, graft uptake and overall quality of life of the patient.^10^ In Pakistan, graft uptake has been reported to be 60% in a study using simple myringoplasty.^13^

Internationally, there is limited data available using PRF to analyze patients with residual tympanic membrane perforations post operatively, for the change in size of perforation and improvement in hearing. Hence, we decided to conduct this research to evaluate the effect of using PRF in Rawalpindi on graft uptake, compare our results with international researches, and lastly, to curtail the deficiencies in local and international literature.

## METHODS

This study was conducted in the Department of ENT of Holy Family Hospital, Rawalpindi, Pakistan, over a period of six months from August 2019 to January 2020. The study was designed as a prospective single blinded randomized controlled trial and was conducted after obtaining an ethical approval from the Institutional Research Forum (IRF), Rawalpindi Medical University (Ref. no. R-29-/RMU, dated 19-09-2018). The participants consisted of 50 patients, who were enrolled after obtaining informed consent, and then divided into two groups consisting of 25 patients each by simple randomization. Randomization was done using a Random Allocation Software^14^ to produce a random selection sequence, and serially numbered envelopes were opened at the time of recruitment.

### Inclusion Criteria

Patients between the ages of 18 and 60 years, having a good Eustachian tube function, absence of cholesteatoma or ossicular discontinuity, having bilaterally dry ears since at least six weeks, a good cochlear function, and lastly, patients agreeing to complete follow up.

### Exclusion Criteria

Patients with co-morbid conditions contraindicating surgery in them, patients having an active ear infection, patients with air-bone gap greater than 50dB and patients who failed to give an informed consent.

### Preoperative Evaluation

Preoperative evaluation included complete history taking and clinical examination of each ear, of the throat and nose. Examination under microscope was done to categorize perforations according to their size as small (less than 50% of total surface area), medium (50-75% of the total surface area) and large (more than 75% of the total surface area). Hearing assessment was performed with pure tone audiometry with masking at 500 Hz, 1000 Hz, 2000 Hz frequencies. Patients included in the study were subjected to routine blood tests and general anesthesia fitness was done.

### Preparation of PRF (Platelet Rich Plasma)

**Fig.1:**
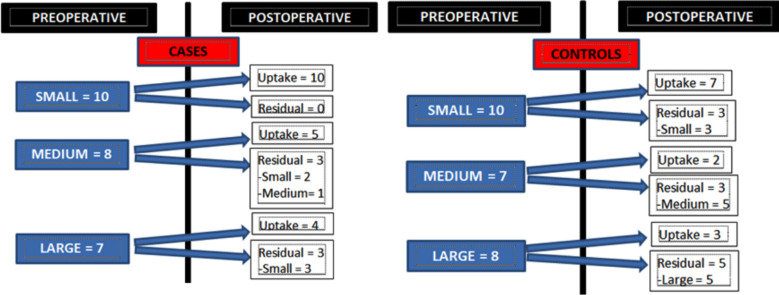
Perforation sizes and Graft Uptake among the cases and controls.

The process of PRF preparation is shown in Fig.1. 10ml of patient’s blood was taken in the Cell’s platelet test tube and centrifuged using a centrifuge machine (CELLS HORIZON 6, made in USA) for seven minutes at 2,300 rpm with 4x15ml swing out head. The resultant product consisted of layers; topmost containing acellular platelet poor plasma, PRF clot in the middle and RBCs at the bottom. The middle layer was then transferred to the Cell’s activation tube, containing the activator (0.03ml CaCl_2_). This activated PRF was then used in our cases.

### Surgical Technique and Procedure

Surgical procedure was performed under general anesthesia, using post-auricular approach. Perforation margins were freshened. Underlay technique was employed and temporalis fascia graft placed. In 25 patients PRF was placed over the graft and in the external auditory canal. While in the remaining 25 patients, plain gel foam was placed. After completion of the procedure, both the groups were prescribed same medications. Patients were instructed to keep their ear dry, avoid forceful blowing of the nose and conditions like flu or constipation. A routine follow-up was recommended. Patients were endoscopically examined consecutively every month for three months, and outcomes were classified on the basis of:

1. Uptake of graft
2. Size of residual perforation (if any)
3. Pure tone audiometry (postoperative audiograms air conduction calculated on 500, 1000, 2000 and 3000 Hz) after three months of surgery.
4. Presence of post-operative complications such as infection signified by post-operative ear discharge.

### Data Collection and Statistical Analysis

Data was collected using a verified and modified Performa by the Institutional Research Forum Committee members. Performa were filled by the principal investigator. Data was entered in SPSS v22.0 (Statistical Package for Social Sciences). The data included age, gender, type of operation, size of the perforation, and condition of the mucosa, graft uptake, post-operative endoscopic findings and complications. Pre-operative and post-operative pure tone audiometry results were used to compute a variable to identify the difference in hearing after the procedure in both groups. Data was expressed as percentages for discrete variables and mean ± SD for continuous variables. Fisher’s 95% confidence intervals (CI) were calculated for the proportions. Pearson’s Chi-square test was used as a test of significance at the 5% level.

## RESULTS

In our study, 50 patients were recruited who ranged between 14 to 46 years of age, mean being 28.62±8.78. Females had preponderance over males, with 28(56%) and 22(44%) participants in each gender group respectively, giving a female to male ratio of 1.27:1. Of all the 50 participants, 20 (40%) had small perforations, 15 (30%) had medium perforations and 15 (30%) had large perforations. These variables had variation when categorized according to the cases and controls as shown by Table-I. Graft uptake was reported to be greater in cases (76%) as compared to the controls (52%), however this was not significant (P=0.070). Post-operative infection was relatively less among cases 8%, compared to 32% among controls. This was statistically significant, (P=0.037). Both the groups were comparable considering the confounding variables such as gender, size of perforation, middle year mucosa condition and ossicles fixation as there wasn’t any significant difference when compared in cases and controls (P>0.137 or more).

**Table-I:** Baseline Characteristics and Results of participants in both groups.

Procedure			

	Simple Myringoplasty (Controls)	Myringoplasty with PRF (Cases)	P-Value
*Age*			0.138 *
Mean	25±8	32±9	
Range	14-40	18-46	
*Gender*			0.388**
Male	10 (40%)	12 (48%)	
Female	15 (60%)	13 (52%)	
*Size Of Perforation*			0.936***
Small	10 (40%)	10 (40%)	
Medium	7 (28%)	8 (32%)	
Large	8 (32%)	7 (28%)	
Middle Year Mucosa Condition			0.235**
Healthy	22 (88%)	25 (100%)	
Congested	3 (12%)	0 (0%)	
*Ossicles Fixed*			0.695**
Yes	2 (8%)	2 (8%)	
No	23 (92%)	23 (92%)	
Post-Operative Infection (P=0.037)*			0.037**
Yes	8 (32%)	2 (8%)	
No	17 (68%)	23 (92%)	
Graft Uptake (P=0.070)*			0.070**
Yes	13 (52%)	19 (76%)	
No	12 (48%)	6 (24%)	

* Independent Sample T Test

** Fischer exact test

*** Pearson Chi Square

Similarly the size of the residual perforation among the cases and controls where uptake of graft did not take place also varied. This was statistically significant (P=0.042). Fig.1 show perforation sizes preoperatively and post-operatively of both cases and controls, signifying that cases had greater graft uptake compared to the controls. There was no residual perforation in 76% of cases and 52% of controls. Moreover, comparatively, size of the perforation was also reduced among the cases.

Hearing improvement was also variable among the cases and controls, being 18.00±6.22 dB and 6.00±6.29 dB (p=0.014). With successful graft uptake there was improvement of 15.66±9.90 dB while without graft uptake was 5.50±5.74 dB (p=0.001). As shown in Table II, hearing generally improved a lot more with the usage of PRF whether there was graft uptake or not.

**Table-II:** Hearing improvement in cases and controls with individual stratification based on uptake.

Overall Improvement in Hearing (dB)	Controls		Cases	

	No Uptake	Uptake	No Uptake	Uptake

Mean	4±6	8±6	9±5	21±8
Range	0-19	0-24	0-18	0-30

## DISCUSSION

In this study, the success of the procedure was indicated by the graft uptake and improvement in hearing among the patients, hence these two variables were regarded as the determinants of overall improvement in quality of life of the patients. In terms of graft uptake, the results of our study signified lesser success rates when compared to international and local studies. Studies involving simple Myringoplasty without PRF showed uptake rates of 64%^11^ and 89.5%^12^ in UK, while in Pakistan a study using underlay technique gave an uptake of 68%,^15^ while another study had uptake of 80%^4^. This was, however, relatively greater than our results of 52% in case of simple myringoplasty. This can be attributed to the poor compliance to follow post-operative instructions among our population.

Studies involving usage of autologous PRF with Myringoplasty have proven to have better results than simple myringoplasty. A study by S. Yadav et al. reported 95% graft uptake and 18.62 dB hearing improvement in PRF group, while 85% graft uptake and 13.15dB hearing improvement in no PRF group.^18^ A similar study done in India by Shanmugam R et al. proved the superiority of using PRF, showing 100% graft uptake with usage of PRF and hearing improvement of 13.75 dB.^19^ Although both the studies had similar technique but sample size was less. Our study had 50 patients in total, making it relatively superior in terms of sampled population. Although graft uptake in our study was 78% in cases, which was relatively less, hearing improvement of 18dB (21dB with uptake and 9 dB without uptake) was more compared to both the studies.

A study done by Anwar et al, in 2015, reported graft uptake of 100% in cases, and 81.25% in controls.^7^ Increased graft uptake in this study, could be explained by the use of PRF in gel form and different graft material employed. While, in our study we used drops of PRF instead of gel form.

A study done by Saeedi et al involving gel foam soaked in PRF gave 66.6% graft uptake.^20^ Hence, different techniques of PRF usage yield different results which is comparable to our study as well. Ultimately it can be concluded that PRF usage in whatever form yields better results than simple myringoplasty. However, more research is still needed to definitely conclude as to which technique of PRF usage is most beneficial.

PRF usage has found to be effective in reducing the incidence of post-operative infection as concluded in a study conducted in Egypt by Fawzy T et al.^21^ An Indian study also showed similar results.^22^ This was comparable to our study where 8% cases and 32% controls reported post-operative infection. The presence of WBCs in high concentrations in our technique for preparing PRF must have had a role against infection with its bactericidal effect.

When comparing the results of usage of PRF among the patients, we saw remarkable improvement in hearing in patients regardless of graft uptake. Though it was relatively more in patients with graft uptake. This was because there was reduction in the size of the perforation resulting in a smaller residual perforation even when complete graft uptake did not take place. Reduction in the size of perforation in residual perforation has not been reported in literature before but it’s, however, comparable to a study done by Anwar et al.^7^ in 2017, in which usage of PRF as a graft resulted in closure of small perforations.

Hence we conclude that not only usage of PRF enhances the healing time and rate of the perforation closure but also reduces the size of perforation resulting in an overall decreased size of residual perforation as well as improved hearing. This is signified in our results where cases with no graft uptake had greater hearing improvement (9dB) compared to controls with no graft uptake (4dB). Our study signifies that PRF aided myringoplasty yields far better results with regards to graft uptake. Hence, leading to improved hearing.

### Limitations of the study

Limitations of our study included difficulty in counselling the patient for a different procedure and short time duration leading to a slightly less sample size.

## CONCLUSIONS

Platelet-rich fibrin aided myringoplasty is a safe and effective technique given that it decreases the size of perforations, decreases post-operative infection rate, increases graft uptake and improves hearing in patients. It gives better results regardless of the technique used to prepare PRF, and should replace simple myringoplasty in order to yield better results. However, studies should be conducted to test which technique for preparation of PRF used in PRF aided myringoplasty is the most effective for the patients.

### Authors’ Contribution:

**NR & MA:** Conceived and designed the study, did the literature review.

**NR:** Did data collection and manuscript editing. Responsible and accountable for accuracy/integrity of the work.

**MSK:** Did statistical analysis, literature review and manuscript drafting.
